# Towards onset prevention of cognition decline in adults with Down syndrome (The TOP-COG study): A pilot randomised controlled trial

**DOI:** 10.1186/s13063-016-1370-9

**Published:** 2016-07-29

**Authors:** Sally-Ann Cooper, Temitope Ademola, Muriel Caslake, Elizabeth Douglas, Jonathan Evans, Nicola Greenlaw, Caroline Haig, Angela Hassiotis, Andrew Jahoda, Alex McConnachie, Jill Morrison, Howard Ring, John Starr, Ciara Stiles, Chammy Sirisena, Frank Sullivan

**Affiliations:** 1 Institute of Health and Wellbeing, University of Glasgow, Mental Health and Wellbeing Group, Gartnavel Royal Hospital, Administrative Building, 1055, Great Western Road, Glasgow, G12 0XH UK; 2 Community Learning Disability Psychiatry, The Gatehouse, Inverurie Hospital, Inverurie, AB51 3UL UK; 3 Institute of Cardiovascular and Medical Sciences, University of Glasgow, McGregor Building, 2nd floor, Western Infirmary, Glasgow, G11 6NT UK; 4 Research and Development NHS Greater Glasgow and Clyde, 1st floor Tennent Institute, Western Infirmary Church Street, Glasgow, G11 6NT UK; 5 Robertson Centre for Biostatistics, University of Glasgow, Boyd Orr Building, Glasgow, G12 8QQ UK; 6 University College London, Bloomsbury Campus, Charles Bell House, 67-73 Riding House Street, London, W1W 7EY UK; 7 Institute of Health and Wellbeing, University of Glasgow, General Practice and Primary Care, 1 Horselethill Road, Glasgow, G12 9LX UK; 8 Department of Psychiatry, University of Cambridge, Douglas House, 18b Trumpington Road, Cambridge, CB2 2AH UK; 9 Alzheimer Scotland Dementia Research Centre, 7 George Square, Edinburgh, EH8 9JZ UK; 10 Scottish Borders Learning Disability Service, Church Street, Earlson, TD4 6HR UK; 11 Gordon F Cheesbrough Research Chair and Director of UTOPIAN, University of Toronto, North York General Hospital, 4001 Leslie Street, Toronto, ON M2K 1E1 Canada

**Keywords:** Alzheimer disease, Dementia, Down syndrome, Neuropsychology, Primary prevention, Simvastatin, Statin

## Abstract

**Background:**

Dementia is very common in Down syndrome (trisomy 21) adults. Statins may slow brain amyloid β (Aβ, coded on chromosome 21) deposition and, therefore, delay Alzheimer disease onset. One prospective cohort study with Down syndrome adults found participants on statins had reduced risk of incident dementia, but there are no randomised controlled trials (RCTs) on this issue. Evidence is sparse on the best instruments to detect longitudinal cognitive decline in older Down syndrome adults.

**Methods:**

TOP-COG was a feasibility/pilot, double-blind RCT of 12 months simvastatin 40 mg versus placebo for the primary prevention of dementia in Alzheimer disease in Down syndrome adults aged 50 years or older. Group allocation was stratified by age, apolipoprotein E (*APOE*) *ε4* allele status, and cholesterol level. Recruitment was from multiple general community sources over 12 months. Adults with dementia, or simvastatin contraindications, were excluded. Main outcomes were recruitment and retention rates. Cognitive decline was measured with a battery of tests; secondary measures were adaptive behaviour skills, general health, and quality of life. Assessments were conducted pre randomisation and at 12 months post randomisation. Blood Aβ40/Aβ42 levels were investigated as a putative biomarker. Results were analysed on an intention-to-treat basis. A qualitative sub-study was conducted and analysed using the Framework Approach to determine recruitment motivators/barriers, and participation experience.

**Results:**

We identified 181 (78 %) of the likely eligible Down syndrome population, and recruited 21 (11.6 %), from an area with a general population size of 3,135,974. Recruitment was highly labour-intensive. Thirteen (62 %) participants completed the full year. Results favoured the simvastatin group. The most appropriate cognitive instrument (regarding ease of completion and detecting change over time) was the Memory for Objects test from the Neuropsychological Assessment of Dementia in Individuals with Intellectual Disabilities battery. Cognitive testing appeared more sensitive than proxy-rated adaptive behaviour, quality of life, or general health scores. Aβ40 levels changed less for the simvastatin group (not statistically significant). People mostly declined to participate because of not wanting to take medication, and not knowing if they would receive simvastatin or placebo. Participants reported enjoying taking part.

**Conclusion:**

A full-scale RCT is feasible. It will need 37 % UK population coverage to recruit the required 160 participants. Information/education about the importance of RCT participation is needed for this population.

**Trial registration:**

ISRCTN67338640.

**Electronic supplementary material:**

The online version of this article (doi:10.1186/s13063-016-1370-9) contains supplementary material, which is available to authorized users.

## Background

Adults with Down syndrome have a very high prevalence of dementia of an Alzheimer disease type; indeed Down syndrome is the commonest cause of early-onset dementia, with 40 % of those aged 50 and over having acquired it [1, 2]. There are no preventative measures routinely available, and hence it is an exceedingly high priority to identify effective interventions.

The amyloid precursor protein (*APP*) gene is over-expressed in Down syndrome (trisomy 21) as it is located on chromosome 21. This leads to amyloid β (Aβ) overproduction, which is thought to be the underlying cause of the high rates of Alzheimer disease [3]. Unlike the general population, adults with Down syndrome develop a relatively ‘pure’ amyloid form of Alzheimer disease, as they have a remarkable resilience to atherosclerosis, being atheroma-free, with low blood pressure, and having low vascular dementia rates [4, 5]. This may be because the cystathionine β synthase (*CBS*) gene is also located on chromosome 21, and so is over-expressed. This results in trans-sulphuration of homocysteine to cysteine, and a lower than normal plasma homocysteine level (the opposite of homocysteinuria, which can be due to CBS deficiency, resulting in hyperhomocysteinaemia and early and aggressive arterial disease). This means that findings produced by trials in the general population cannot be assumed to be generalisable to people with Down syndrome.

Statins (microsomal 3-hydroxy-3-methylglutaryl coenzyme A reductase inhibitors) are plausible agents to reduce the risk of Alzheimer disease. They have pleiotropic effects including potentially increasing brain amyloid clearance by the low-density lipoprotein receptor (LDLR) family of proteins. Experimental animal models support this [6, 7]. Foetal brain LDLR activity has been shown in vitro to be increased significantly, especially in astrocytes, by statin treatment [8].

In humans, there has been little study of statins in adults with Down syndrome. In the single observational Down syndrome study that we could identify, statin use and incident dementia was prospectively investigated over a 5-year period in 123 participants aged 40 years and older [9]. The participants on statins were found to have less than half the risk of incident dementia, and persons with Aβ42 in the middle or highest range were more than twice as likely to have incident dementia [10]. We have identified no randomised controlled trials (RCTs) of statins in people with Down syndrome.

Regarding the general population, Alzheimer disease is less prevalent in populations with low blood cholesterol and diets that are low in fat and cholesterol [11, 12]. Several case-control studies have reported a lower risk of dementia among statin users [13–19]. Prospective cohort studies have found that statins predicted reduced incidence of dementia or slower cognitive decline [19–26], or reduced hospitalisation due to dementia [27]. Other studies reported no associations of statin use with Alzheimer disease [18, 28, 29], but did not distinguish Alzheimer disease from the vascular dementias, or had unusually high baseline educational levels. A further complication is whether the observed period of statin exposure was during the at-risk period.

A recently updated Cochrane review found only two published statin RCTs for the primary prevention of dementia in the general population [20]: the MRC/BHF Heart Protection Study (HPS) [30] and the Prospective Study of Pravastatin in the Elderly at Risk (PROSPER) study [31]. Neither reported benefits. However, their study participants were selected specifically for having vascular disease and/or vascular risk factors, so are not directly relevant to adults with Down syndrome who have Aβ overproduction. Pravastatin, used in PROSPER, acts on vascular targets but not any within the central nervous system as it is hydrophillic; hence, theoretically it is unlikely to be effective in preventing dementia in people with Down syndrome. Conversely, simvastatin is lipophilic and crosses the blood-brain barrier. Smaller studies including ‘Alzheimer disease high-risk groups’ have found benefits at 4 months and 6 months [32, 33], although not consistently so [34]. A small, secondary prevention RCT showed promising results of atorvastatin in slowing cognitive decline at 6 and 12 months [35], whereas two larger RCTs did not [36, 37]. However, theoretically, statins may be more effective in primary than in secondary prevention. Authors of two more reviews concluded that trials are indicated specifically when Alzheimer disease is due to amyloid overproduction [38, 39], and a recent systematic review concluded that there is an absence of well-powered RCTs for most cognitive outcomes of statins and that larger and better-designed studies are needed [40]. However, this general population literature is unlikely to be relevant to the atheroma-free Down syndrome population.

Adults with Down syndrome have pre-existing cognitive deficits and, therefore, existing norms on neuropsychological test instruments designed for the general population do not apply for them, and these instruments are also typically too complicated for adults with Down syndrome to complete, resulting in a ‘floor effect’. There have been previous studies which measured changes with age using adapted or specially devised assessment tools [2, 41, 42], but most are limited by small sample sizes and are not longitudinal. Measures of cognitive function may be a more accurate and sensitive measure of decline than caregiver-reported changes in adaptive function, particularly given staff-turnover amongst paid carers, meaning that different carers report at different times and may not know the person well. Hence, further delineation of which cognitive tests are most sensitive is important.

Further details on the background to this study have been previously reported in a protocol paper [43].

### Study aims

The study aims were to:

1. Acquire data to design a full-scale, multi-centre RCT of simvastatin for the primary prevention of dementia in Alzheimer disease in older adults with Down syndrome
2. Test recruitment and retention strategies to inform future trials with this population
3. Determine the best instruments to use in future studies measuring cognitive decline in adults with Down syndrome
4. Investigate mechanisms, using Aβ42/Aβ40 measurements as a putative surrogate biological marker

### Research questions

The research questions were to identify:

1. Trial recruitment/retention rates and recruitment sources
2. Rates of tolerability/safety of simvastatin 40 mg at night
3. The most sensitive instruments to detect early cognitive decline with least floor effect with Down syndrome adults
4. The perceptions of adults with Down syndrome and their carers on deciding whether to participate, and to be randomised, and their experience of the assessments
5. The distributions of the primary (cognitive decline) and key secondary (adaptive behaviour, general health, quality of life) outcome measures that would be used in a definitive RCT, and the sample size implications of these distributions
6. Whether Aβ42/Aβ40 is a biomarker for cognitive decline
7. Whether the results support proceeding to a full RCT

## Methods

Study methods have previously been reported in more detail in the protocol paper [43]. A Consolidated Standards of Reporting Trials (CONSORT) checklist is provided at Additional file 1.

### Study design

TOP-Cog was a feasibility and pilot double-blind RCT of 12 months simvastatin versus placebo for the primary prevention of dementia. It included a qualitative study, analysed using the Framework Approach [44], to determine motivators and barriers to recruitment and the experience of study participation. We made no methodological changes after registration.

### Setting

The setting was the general community of Scotland, UK, specifically the health board areas of Greater Glasgow and Clyde, Lothian, Tayside, Lanarkshire and Borders. Recruitment was from multiple sources within the general community, Down Syndrome Scotland, Local Authorities, and National Health Service clinical services.

### Participants

#### Inclusion criteria

1. Down syndrome
2. Aged 50 years and over

#### Exclusion criteria

1. No consent obtained
2. Unable to comply with the protocol, including providing blood or saliva for baseline apolipoprotein E (*APOE*) *ε4* measurement, and venous or capillary blood for cholesterol measurement
3. Dementia at baseline (as the study is investigating primary prevention)
4. Diabetes (as this is an indication for a statin prescription)
5. Clinically evident atherosclerotic disease (as this is an indication for a statin prescription)
6. Being at risk for cardiovascular disease (as this is an indication for a statin prescription)
7. Liver disease
8. Chronic renal insufficiency
9. Being prescribed a statin or medicines that are listed as contraindicated with simvastatin in its summary of product characteristics, plus miconazole oral gel
10. Having previously had a statin-related serious adverse event
11. Unable or unwilling to avoid consumption of grapefruit juice
12. Alcohol use of over 21 units/week for men, or over 14 units/week for women

### Intervention and comparison

The intervention was one over-encapsulated simvastatin 40 mg tablet at night for 12 months, compared with one capsule of placebo at night for 12 months. Tablet and placebo were identical in appearance and similar in weight.

### Outcome measures

#### Primary measures

1. Recruitment and retention:
  - Numbers screened and recruited each month
  - The proportion of participants retained 12 months after randomisation
  - The number of participants recruited per base general population size

#### Secondary measures

1. Adverse and serious adverse events
2. Cognitive test battery to measure cognitive decline:
  - Memory for Objects from the Neuropsychological Assessment of Dementia in Individuals with Intellectual Disabilities (NADIID) battery [41]
  - Selective Attention Cancellation Test [42]
  - Pattern recognition memory from the Cambridge Neuropsychological Test Automated Battery (CANTAB) [45]
  - Cats and Dogs test [46]
  - Tower of London Test (a test of frontal lobe executive functioning, recently adapted by our group for intellectually disabled adults) [47]
  - Cued Recall Test [48]
  - Category Fluency Test (asking participants to think of as many animals as they can in 1 minute)
  - Story Recall Test (adapted from the Rivermead Behavioural Memory Test for Children) [49]

Further information on the cognitive test battery is included at Additional file 2.

3. Key themes from semi-structured interviews
4. Estimate of the sample size needed for a full RCT from the cognitive test battery; EuroQol 5-Dimension Questionnaire (EQ-5D) score, recently reviewed for the intellectually disabled population [50]; Townsend Scale score [51]; Adaptive Behaviour Scale – Residential and Community score (ABS) [52]
5. Aβ40/Aβ42 levels
6. The size of the geographical recruiting area and hence the likely associated costs of a full trial, and the sensitivity of, and floor effect on, the cognitive measures

### Group allocation and blinding

Participants were randomly assigned to either simvastatin or placebo, stratified by age (50–54 years or 55 years and over), apolipoprotein E (*APOE*) *ε4* allele status (presence of an *ε4* allele or not), and cholesterol level (below 5 mmol/l or 5 mmol/l and over). These stratifications were made in case of a differential effect on outcome; the absolute level taken to define high and low cholesterol is of course an arbitrary decision; as is the absolute age level to delineate younger and older participants. Regarding age, we aimed to balance the increased incidence of dementia with age against the smaller pool of potential recruits. Since we did not know a priori what the effect sizes we might detect were, difficulty in recruitment, attrition, etc. at different ages, we predefined an age stratification at what we estimated would be the mid-point of the range of those recruited. To ensure blinding to both *APOE* status and group allocation, the researcher notified the Robertson Centre for Biostatistics (RCB) of the participant’s study number and age via a web portal. The laboratory then notified the RCB of the participant’s *APOE* status and cholesterol level via the portal. The RBC then notified pharmacy of group allocation, and generated an email to the researcher notifying that randomisation has taken place, and the medication pack number assigned. Pharmacy then dispensed the medication by posting it to participants on a 3-monthly basis from a central pharmacy (rather than expecting participants/carers to collect it).

### Process and assessments

Invitations were distributed to potential participants from a variety of sources. An initial telephone call was made to individuals who replied to an invitation, to discuss study participation. Interested individuals were then screened for inclusion/exclusion criteria and a researcher took consent from the person or, for people who did not have decision-making capacity to consent for themselves, consent was taken from their legal representative, in keeping with the Medicines for Human Use (Clinical Trials) Regulations, 2004. At the initial home visit, baseline data were collected on cognitive function, adaptive function, health, and quality of life. Also taken at baseline were venous or capillary blood samples for *APOE* and cholesterol levels, liver function tests, creatine kinase and thyroid function tests, and if possible, venous blood for baseline measures of Aβ40/Aβ42. Sensory assessments were also conducted to check the extent to which they could use some of the cognitive test instruments. Participants were then randomised and drug/placebo prescribed. After 6–12 weeks a safety visit was conducted, and blood was taken for alanine transaminase, aspartate transferase, and creatine kinase. Each participant/carer was then telephoned at 3, 6 and 9 months to enquire specifically about adverse events and any changes to supports. Twelve months after randomisation, cognitive assessments were repeated at a home visit, as was assessment of adaptive function, health, quality of life, service use, and adverse events. Additionally, at 12 months, blood was taken for cholesterol, liver function tests and thyroid function tests, and if possible, venous blood for Aβ40/Aβ42. All biochemical analyses were conducted in a core Clinical Pathology Accredited laboratory. Serum was stored frozen at −80 °C. Baseline and 12-month samples were analysed in the same assay.

For the nested qualitative study, home visits were undertaken by the researcher 6 months after recruitment started. Attempts were also made to interview persons who declined to participate in the RCT. Interviews were jointly with the person with Down syndrome and their carer, reflecting the likely way that decisions were taken about participation in the study. Topic guides were used, but planned to be flexible because interviews with people with intellectual disabilities are sometimes challenging and the interviewer must be patient and sensitive to the person’s needs. Interviews were tape recorded and transcribed verbatim.

### Power calculation

Recruitment feasibility was planned to be assessed by the number of people identified/1,000,000, the percentages of those contacted who would like to participate, and of those, the percentage who were eligible. For example, if 200 individuals were contacted, and 100 agree to be screened, of whom 60 were eligible for randomisation, then the overall recruitment rate would be 30 % with a 90 % confidence interval (CI) of 25–36 %; sufficiently accurate to allow planning for a larger RCT. Similarly, if 50 of the 60 participants completed the 12 month follow-up, the retention rate would be 83 % with a 90 % CI of 73–91 %.

The variance of the rate of cognitive decline was planned to be estimated to calculate the sample size required for a definitive RCT; the precision of this estimate is a function of the sample size in this pilot study. We decided that 50 participants providing 12-month outcome data was an appropriate sample size to provide a variance estimate that is reasonably precise, without recruiting an excessively large sample. With 50 subjects, a 90 % CI for the variance would have a width of approximately 70 % of the estimated variance. A smaller sample size would increase the uncertainty in the variance estimate, whereas to obtain a more precise estimate could require considerably more participants, e.g. to half the width of the 90 % CI for the variance would require 180 subjects, which would have been too large for a pilot designed to show feasibility of an RCT.

For the qualitative study, we did not know in advance how many interviews would be necessary to reach saturation, but planned to interview 10 dyads of participants with Down syndrome/their carer, and attempted to also recruit 10 dyads of adults with Down syndrome/their carer who chose not to participate in the pilot RCT. Sampling was purposive to include both paid carers and family carers, and people with Down syndrome with a range of ability levels.

### Types of analyses

Results were analysed on an intention-to-treat basis. Continuous variables are summarised as mean and standard deviation; categorical variables as frequencies and percentages. Outcome variables are summarised at baseline, at 12 months follow-up, and as the change over time (follow-up minus baseline). For each outcome, a linear regression model was fitted to estimate the treatment effect (simvastatin minus placebo), where the outcome variable was the follow-up measurement, and the predictor variables were randomised treatment group, the baseline value of the outcome measure, and the variables used to stratify the randomisation, namely age (50–54 years or 55 years and over), *APOE ε4* allele (presence of an *ε4* allele or not), and cholesterol level (below 5 mmol/l or 5 mmol/l and over). Where the linear model was a poor fit to the data, outcomes were collapsed to binary, and binary logistic regression was used. The estimated treatment effect and 90 % confidence limits were divided by the standard deviation of the outcome at baseline in the whole population, giving an estimated effect size and 90 % CI for each outcome where a linear model was a reasonable fit to the data.

All statistical analyses were conducted using SAS software v9.3 (SAS Institute, Cary, NC, USA).

The qualitative interviews were analysed using the Framework Approach to identify similarities and differences in the data and draw descriptive or explanatory conclusions around themes. After verbatim transcription of the interviews, the transcripts were read and reread by CS and JM to familiarise themselves with the data. Codes were applied to important phrases separately by the two researchers and, after meeting to discuss the codes, they were grouped into categories and summarised. Several meetings then took place between CS and JM to interpret the data [44].

### Ethics

The study was approved by the Multi-centre Research Ethics Committee – Scotland A (11/AL/0200), and the Medicines and Healthcare Products Regulatory Agency (EudraCT number 2011-001564-21). NHS research and development site approvals were gained from NHS Greater Glasgow and Clyde, Lothian, Tayside, Lanarkshire, and Borders. Approvals were also required and gained from Edinburgh Council, and Glasgow City Council. South Lanarkshire Council refused ethical approval. Other local authorities did not require ethical submission. The sponsor’s protocol for assessing and reporting adverse events in clinical trials of investigative medicinal products was adhered to.

## Results

### Trial recruitment/retention rates and recruitment sources

We recruited and randomised 21 participants over a 12-month period.

Scotland’s census, 2011 data reveals the base population size of the area we recruited from was 3,135,974; including 2,579,698 adults. Hence, we expected there to be 387 adults with Down syndrome aged 50 years and over [53]. For this age group, we would expect about 40 % to have dementia [1, 2], our major exclusion criteria, leaving 232 eligible to be considered for the trial. We identified 217 adults initially thought to be eligible, but on further consideration, 36 had dementia, hence we identified 181 potential participants; 78 % of the expected eligible population.

We invited the potential participants, and after assessing inclusion and exclusion criteria, randomised 21 of these 181 individuals (11.6 %). Their characteristics were balanced in the two arms (Table 1). Recruitment, therefore, was 21 participants from a population size of 3,145,974 (or 6.7/1,000,000 whole population), and by area was:

- 9 in Greater Glasgow and Clyde (7.4/1,000,000)
- 6 in Lothian (10.8/1,000,000)
- 3 in Tayside (7.3/1,000,000)
- 2 in Lanarkshire (3.5/1,000,000)
- 1 in Borders (8.8/1,000,000)

**Fig. 1:**
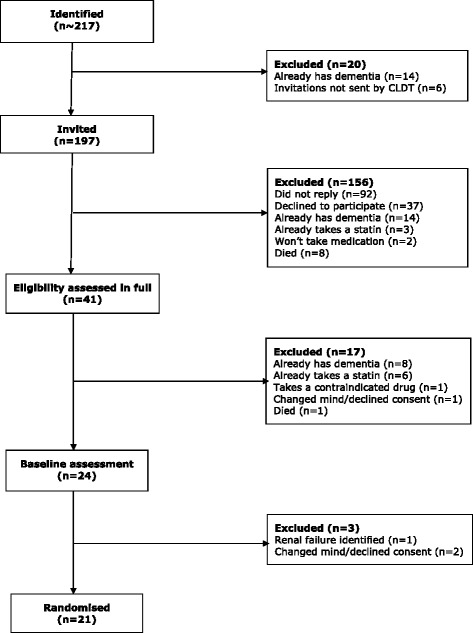
Recruitment flowchart

**Fig. 2:**
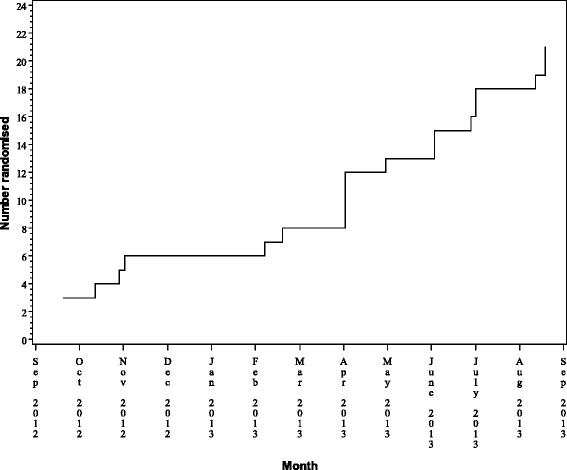
Cumulative randomisation over time

Figure 1 shows the recruitment flowchart. Figure 2 shows cumulative randomisation over time.

**Fig. 3:**
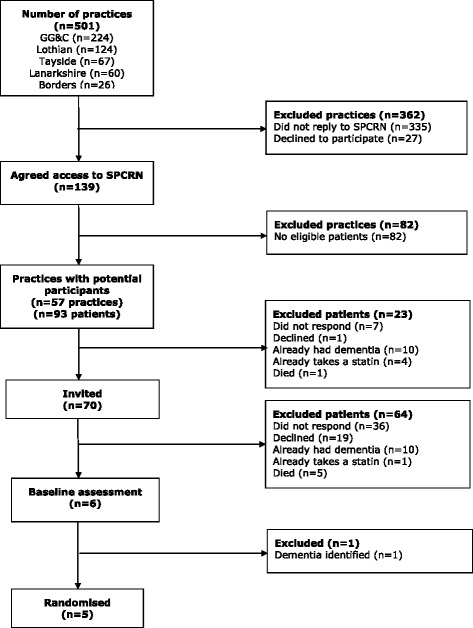
Recruitment via the Scottish Primary Care Research Network

**Fig. 4:**
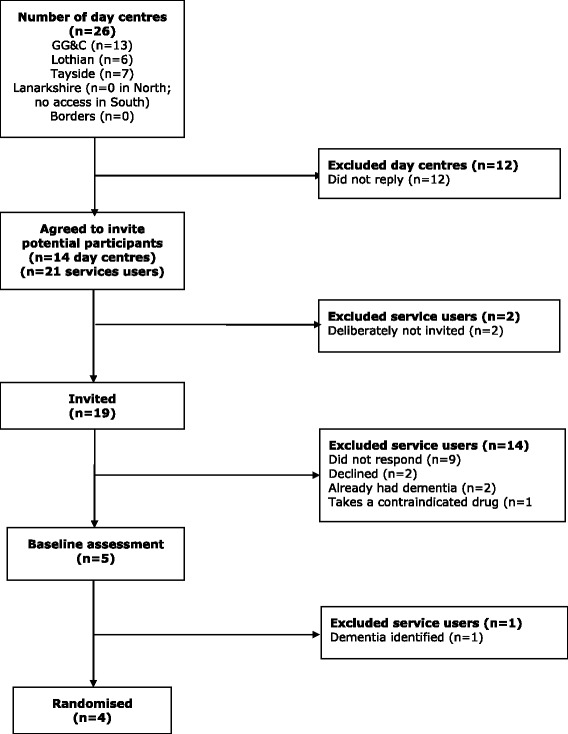
Recruitment via learning disabilities day centres

We did not recruit any participants via organisations providing support for people with learning disabilities, despite attempting to. Recruitment sources were:

- Scottish Primary Care Research Network – 5 (Fig. 3)
- Learning disabilities day centres – 4 (Fig. 4)
- Learning disabilities psychiatrists – 4
- Other learning disabilities health professionals – 3
- Previous participants of the chief investigator’s research – 3
- Down Syndrome Scotland – 2

Twenty additional individuals responded to the invitation expressing interest to participate, but were excluded. They were identified via:

- Scottish Primary Care Research Network – 5
- Learning disabilities day centres – 3
- Learning disabilities health professionals – 1
- Previous participants of the chief investigator’s research – 10
- Down Syndrome Scotland – 1

**Table 1:** Baseline demographics

Characteristic	Overall *N* = 21	Control *N* = 11	Intervention *N* = 10
Age (years)			
Mean (SD)	54.15 (3.10)	53.67 (3.16)	54.68 (3.10)
Sex			
*N* (%) male	11 (52 %)	6 (55 %)	5 (50 %)
*N* (%) female	10 (48 %)	5 (45 %)	5 (50 %)
*APOE*			
*N* (%) *ε3/2*	2 (10 %)	1 (9 %)	1 (10 %)
*N* (%) *ε3/3*	13 (62 %)	7 (64 %)	6 (60 %)
*N* (%) *ε4/3*	6 (29 %)	3 (27 %)	3 (30 %)
Ability			
*N* (%) mild ID	4 (36 %)	4 (36 %)	0 (0 %)
*N* (%) moderate ID	7 (33 %)	3 (27 %)	4 (40 %)
*N* (%) severe ID	9 (43 %)	4 (36 %)	5 (50 %)
*N* (%) profound ID	1 (5 %)	0 (0 %)	1 (10 %)

*APOE* apolipoprotein E gene, *ID* intellectual disability

Thirteen (61.9 %) of the 21 participants completed the trial in full. Table 2 reports the reasons why eight participants did not complete the 12 months of medication; three of these eight completed the 12-month post-randomisation interview. Hence, the 12-month interviews were completed by seven (70 %) in the simvastatin group and nine (82 %) in the control group. A CONSORT flow diagram is included in Additional file 3.

**Table 2:** Reasons for not completing the full year of medication

Reason	Group allocation
Skin rash and GP advised to stop medication	Simvastatin
Weight loss, hitting leg – symptoms preceded taking the medication, but relative thought possibly worse so stopped medication	Simvastatin
Onset of thyroid dysfunction and carer chose to stop medication	Placebo
History of episodic diarrhoea. Diarrhoea after commencing medication so relative stopped medication	Simvastatin
GP prescribed a statin	Placebo
Paid carer withdrew consent	Simvastatin
Dementia diagnosed, and relative stopped medication	Placebo
Participant changed their mind	Placebo

By extrapolation, to recruit 160 into a full-scale RCT, to retain 99, a population size of approximately 23.9 million is required.

### Tolerability/safety

There were no serious adverse events, and no adverse events definitely attributable to medication.

### Most sensitive instruments to early cognitive decline with least floor effect

Table 3 shows how many participants were able to complete the cognitive tests. Table 4 shows the distribution (mean and standard deviation) for outcome measures at baseline, 12 months, and change over time, as well as treatment effect estimates expressed as effect sizes with 90 % CIs. Effect size estimates have been reversed where necessary, so that a positive effect can be viewed as a benefit for the simvastatin group.

**Table 3:** Completion of cognitive tests

Test	Unable to complete at baseline *N* = 21	Unable to complete at 12 months *N* = 16
NADIID Memory for Objects test	1	1
Selective Attention Cancellation Test – overall score	6	3
CANTAB pattern recognition test – % correct	10	3
Cats and Dogs switching condition – time taken	6	5
Cats and Dogs switching condition – errors	6	5
Tower of London Test (revised) – total score	1	3
Cued Recall Test – overall score	5	2
Category Fluency Test – total number of words correct	2	2
Category Fluency Test – total number of repeated words	2	2
Category Fluency Test – number of errors	2	2
Story Recall Test – free recall total score	3	6
Story Recall Test – cued recall total score	3	6

*CANTAB* Cambridge Neuropsychological Test Automated Battery, *NADIID* Neuropsychological Assessment of Dementia in Individuals with Intellectual Disabilities

**Table 4:** Mean and standard deviation (SD) on cognitive tests at baseline, 12 months, and change over time

		Placebo			Simvastatin			Effect size
		Baseline	12 months	Change	Baseline	12 months	Change	
NADIID								
	Memory for Objects test	5.7 (2.3)	4.9 (2.6)	−0.8 (1.2)	4.0 (2.4)	5.3 (2.7)	1.3 (1.0)	1.0 (0.4, 1.6)
Selective Attention Cancellation Test								
	Overall score	17.0 (9.3)	18.8 (11.6)	1.8 (14.1)	25.3 (25.0)	21.7 (8.4)	−3.7 (22.6)	−0.4 (−2.1, 1.3)
CANTAB pattern recognition memory								
	% Correct	61.5 (23.5)	67.3 (17.4)	5.8 (6.7)	56.6 (12.3)	57.0 (12.2)	0.4 (13.4)	−0.9 (−1.9, 0.1)
Cats and Dogs switching condition								
	Time taken	58.8 (36.3)	47.0 (31.5)	−11.8 (26.3)	40.5 (22.0)	44.0 (10.9)	3.5 (27.4)	0.05 (−1.3, 1.4)
	Number of errors	4.1 (5.3)	7.0 (8.2)	2.9 (8.6)	11.0 (7.6)	7.8 (7.5)	−3.3 (6.5)	–^a^
Tower of London Test (revised for learning disabilities)								
	Total score	21.7 (8.5)	24.8 (13.6)	3.1 (11.1)	20.7 (8.0)	20.3 (11.7)	−0.3 (18.4)	−0.7 (−2.3, 0.86)
Cued Recall Test								
	Total score	34.1 (4.5)	28.7 (13.0)	−5.4 (12.7)	21.2 (12.7)	12.8 (14.2)	−8.3 (13.9)	–^a^
Category Fluency Test								
	Number correct	12.1 (4.2)	9.2 (3.9)	−2.9 (4.4)	8.0 (4.2)	8.2 (6.1)	0.2 (3.5)	0.3 (−1.1, 1.7)
	Number repeated	0.7 (1.1)	1.0 (1.1)	0.3 (0.9)	2.6 (1.7)	1.6 (1.1)	−1.0 (1.2)	0.5 (−0.2, 1.3)
	Number of errors	0.3 (0.5)	0.1 (0.3)	−0.2 (0.7)	0.0 (0.0)	0.2 (0.5)	0.2 (0.5)	–^a^
Story Recall Test								
	Free recall	5.8 (3.7)	3.6 (3.3)	−2.1 (3.2)	5.0 (6.3)	3.0 (4.2)	−2.0 (3.7)	−0.4 (−1.2, 0.4)
	Cued recall	4.4 (2.7)	3.9 (3.0)	−0.5 (2.9)	2.4 (4.3)	2.6 (3.7)	0.2 (2.3)	−0.6 (−1.8, 0.7)

Effect size reported as estimated between-group difference, adjusted for baseline score and stratification variables, divided by baseline SD in whole population, and reversed where necessary so that a positive effect size corresponds to a benefit for simvastatin group

^a^These are logistic models with decline versus no decline as the response; therefore, no effect size is given

*CANTAB* Cambridge Neuropsychological Test Automated Battery, *NADIID* Neuropsychological Assessment of Dementia in Individuals with Intellectual Disabilities

The tests that most people could complete, and which showed decline over time without floor effect, were the number of objects correct in the NADIID Memory for Objects test [41], the Category Fluency Test, and the Cued Recall Test [48]. The Tower of London Test (revised for this population) [47] was also off the floor, being completed by all but one person, but showed less change. The Selective Attention Cancellation Test [42] showed some utility. These tests represent a mix of memory, executive function, and attention tests and together appear the most appropriate primary and secondary cognitive outcome measures for a full-scale RCT.

The Cats and Dogs test [46], Story Recall Test [49], and CANTAB pattern recognition memory test [45] had poor utility in terms of participant completion.

### Perceptions of adults with Down syndrome/carers on deciding whether to participate, accept randomisation, and their experience of assessments

Semi-structured interviews were conducted with 10 participant/carer dyads who participated in the RCT, and four who had declined (consent was subsequently withdrawn from one). An additional five who had declined provided written responses to the topic guide, but were unwilling to be interviewed.

The analysis identified seven main themes; the numbers in inverted commas reference the supporting quotations which are provided in Additional file 4.

- Value of research. All RCT participants felt research to be worthwhile and important ["^1^, ^2^"]. They felt it is a way to increase our understanding and improve what we already know ["^3^, ^4^"]. Those who declined to participate also acknowledged that research has value ["^5^"]. Some who declined felt they did not know enough about research ["^6^, ^7^"].
- Information sheets are useful, and an additional resource. Most participants felt that the information sheets were useful and had just enough information ["^8^–^10^"]. Additionally, they saw them as an added resource to draw upon for information on dementia and Down syndrome ["^11^, ^12^"]. All RCT participants, and those who declined, felt the source of the information sheets would not have impacted upon their decision whether or not to participate ["^13^–^16^"].
- Preventing the onset of dementia was the primary motivation for deciding to participate. All participant/carer dyads expressed concern about developing dementia ["^17^–^20^"]. Preventing the onset of dementia was the primary motivator in deciding to participate ["^21^–^23^"]. Additionally, some participants believe that helping others is a reason to participate ["^24^, ^25^"].
- Taking tablets was the most common reason for declining to participate. All who declined RCT participation cited taking tablets as the main reason ["^26^–^28^"]. Some expressed concern over potential side effects ["^29^, ^30^"]. Not knowing whether they would be taking a placebo or statin was also a participation deterrent ["^31^, ^32^"].
- Tablets are acceptable if they are necessary. Overall, most participant/carer dyads did not have a problem with taking tablets if they are necessary. A number were already taking tablets ["^33^–^36^"]. Though sometimes difficult, the majority understood the need for being blind to placebo or statin ["^37^–^40^"] – it just influenced their decision
- Taking part in the RCT is a positive experience. Participants enjoyed taking part in the RCT ["^41^–^43^"]. Some felt it provided an opportunity to learn new things about their relative ["^44^, ^45^"]. The majority stated that it was unobtrusive ["^46^, ^47^"] and the assessments interesting ["^48^"].
- Lack of accessible information on Down syndrome and dementia. Almost all participants felt they lacked access to research and information regarding Down syndrome and dementia ["^49^–^52^"]. Many participants were not told about the risk of developing dementia ["^53^, ^54^"].

Additionally, at the point of declining, 19 people indicated why:

- Not wanting medication – 6
- Wanting to keep things normal – 3
- Too much going on – 3
- Thinking the study was illegal – 2
- Not wanting to ‘go down that road’ – 1
- Carer not wanting to upset relative with Down syndrome – 1
- Viewing it as disgusting – 1
- Thinking it was too confusing – 1
- Concern about challenging behaviour – 1

Some general practices contacted via the SPCRN declined to participate on the grounds that they had very few patients who were likely to be eligible.

### Distributions of the outcome measures, and sample size implications

The NADIID Memory for Objects test scores at baseline and 12 months were on average near the middle of the possible range (0–10) and showed a difference between groups, due to an increase in the treatment group and decrease in the control group (Table 4). The reduced standard deviation of the change over time compared to the baseline and 12-month scores indicates a strong correlation over time, suggesting improved efficiency from an analysis adjusted for baseline scores. The CI for the effect size estimates with this outcome suggests the true effect size from simvastatin treatment to be at least 0.4.

Point estimates of the effect sizes for other cognitive measures were mixed, with some positive (in favour of simvastatin) and others negative. However, none showed a statistically significant effect (in either direction) and all had CIs for the effect size estimate that were consistent with true effect sizes of at least 0.4, with the exception of the CANTAB pattern memory recognition score, which had the greatest floor effect. Standard deviation estimates for the changes over time with other cognitive measures were of a similar magnitude to individual measures, suggesting some correlation over time, but less marked than with the NADIID Memory for Objects test.

These data suggest that the NADIID Memory for Objects test may be the best single measure for future use. A clinically relevant 1-point difference between groups at 12 months would be an effect size of 0.42, relative to the standard deviation of scores at baseline, but in relation to the standard deviation of changes over 12 months, would be an effect size of 0.67. A study with 48 participants in each group would be required for 90 % power to detect such a difference, i.e. 160 participants at baseline with 62 % retention.

The cognitive test battery, as expected, had more utility than the ABS (proxy-report on skills and function), which had poor sensitivity to change over the 12 months (Table 5). Of the 10 ABS domains, the language domain showed significantly better outcomes for the intervention compared to the control group (*p* = 0.0168), but other domains did not appear sensitive to change over this 1-year period. The cognitive test battery also showed more utility than the EQ-5D. The Townsend Scale (general health) better distinguished groups than the ABS or EQ-5D (Table 5).

**Table 5:** Mean and standard deviation (SD) for cholesterol, quality of life, and functional scores at baseline, 12 months, and change over time

	Placebo			Simvastatin			Effect size
	Baseline	12 months	Change	Baseline	12 months	Change	
Cholesterol	5.3 (0.8)	5.4 (0.7)	0.1 (0.4)	5.1 (0.5)	4.7 (0.7)	−0.4 (0.9)	1.5 (−0.8, 3.9)
EQ-5D health utility	0.81 (0.29)	0.75 (0.29)	−0.06 (0.20)	0.54 (0.38)	0.68 (0.25)	0.14 (0.38)	0.1 (−0.7, 0.8)
ABS total score	224 (39)	214 (38)	−10 (12)	177 (50)	170 (56)	−7 (20)	−0.1 (−0.4, 0.2)
Townsend Scale total score	8.2 (3.0)	8.1 (3.4)	−0.1 (1.3)	9.3 (4.3)	11.3 (3.3)	2.0 (3.4)	0.7 (0.0, 1.4)

Effect size reported as estimated between-group difference, adjusted for baseline score and stratification variables, divided by baseline SD in whole population, and reversed where necessary so that a positive effect size corresponds to a benefit for simvastatin group

*ABS* Adaptive Behaviour Scale, *EQ-5D* EuroQol 5-Dimension Questionnaire

### Aβ42/Aβ40

Fourteen people donated blood at both baseline and 12-month follow-up for analysis of Aβ42/Aβ40. After adjusting for stratification factors, change over the 12 months was less for the simvastatin group by 24.4 pmol/l for Aβ40 (*p* = 0.122), by 0.26 pmol/l for Aβ42 (*p* = 0.868), and by 0.02 for the Aβ42/Aβ40 ratio (*p* = 0.809).

### Do the results support proceeding to a full RCT?

The rationale for proceeding to a full RCT remains. Based on the cognitive test results, we propose the NADIID Memory for Objects test as the primary outcome measure and found no reason not to proceed to a full-scale RCT. There were no safety reasons identified for not proceeding to a full-scale RCT.

The results show that a sample size of 160 is needed in a full-scale RCT, to retain approximately 100 in the final analysis at 12 months. This will require recruitment across a population of 23.9 million, or 37 % of the UK (population = 64.1 million). We therefore conclude a that full-scale RCT is feasible in the UK, and affordable given the potential benefits.

## Discussion

This study has provided valuable data essential to design a much needed full-scale RCT. It is now possible to design a RCT with a realistic recruitment strategy, and to seek an appropriate level of funding to implement it.

Even with the general population, recruitment is often challenging in trials; a study of 114 UK trials funded by the Medical Research Council (MRC) and National Institute for Health Research - Health Technology Assessment programme (HTA) found that only 31 % recruited successfully, whilst 45 % recruited less than 80 % of intended, and more than half required an extension [54]. A recent Cochrane review synthesised the evidence around strategies to improve recruitment to RCTs [55], and concluded that trialists should include evaluations of their recruitment strategies. Studies with people with learning disabilities are considerably more challenging than general population studies; indeed they are notoriously difficult to recruit into [56–58], and RCTs to investigate medications are rare with this population [59]. Hence, this study was essential prior to designing a full-scale RCT. Additionally, we did not know the proportion of the potentially eligible population we would identify in order to invite them, and we now have this data.

Most participants were recruited via health professionals and day services. Time invested in meeting managers of support-providing organisations did not translate into recruitment. The study was boosted by the Scottish Primary Care Research Network adoption, although labour-intensive for the network as it involved approaching all practices, rather than the small number of research-friendly practices that the network usually work with. This may become less onerous as plans to enable remote access to general practitioner data for research are spreading throughout the UK [60]. As some practices declined to participate because they had few eligible participants, a full-scale RCT should, in its initial letter to practices, more prominently highlight that each practice will have few, and hence the need to recruit through as many practices as possible.

Whilst there might be recruitment, recruitment source, and retention variation across the UK, we consider the study findings highly informative, particularly as they spanned five health boards, rather than a single service. In view of possible variability, a full-scale RCT should include an internal recruitment pilot in several UK sites.

The nested qualitative study findings largely endorse our approaches, rather than generating new ideas to improve recruitment and retention. The main reason people did not participate was concern about taking the medication, and not knowing which group they would be randomised to. The study was about prevention, rather than treatment, which might have had a bearing, given that people were taking medication for other conditions when they needed it. It also seems some people with Down syndrome and some paid carers are not aware of the high dementia risk. There are likely to be other reasons for non-participation, as this information only comes from people who were willing to participate in the qualitative study. It therefore largely provides information relevant to participation in medication trials, rather than other types of research. In view of this information, trial participation rate, and other findings, e.g. failure to gain ethical approval in South Lanarkshire, we consider that there is a considerable need for publicity and education on the reasons for and benefits of conducting RCTs with this population. Such a ‘campaign’ to engage support providers, service users, relatives, and professionals might be beneficial prior to embarking upon a full-scale RCT, or as an early component of it [54]. Another option might be to consider alternative trial designs, such as a patient preference trial design; however, negative as well as positive considerations are needed in such choices, for example, a patient preference design may increase the size and cost of a trial which is already likely to be costly.

Whilst several Down syndrome cohorts exist, to our knowledge, this is currently the largest worldwide, at age 50 and over, to have reported detailed cognitive testing longitudinally. Previous studies have used cross-sectional measurements of memory, attention, and executive functioning to investigate cognitive decline with ageing, but there are no longitudinal reports with more than a handful of participants aged 50 and over. Hence, this study is a major step forward in delineating the most useful cognitive test battery to show change over a short 12-month period for this age group. As expected, cognitive performance was more sensitive than carer-reported adaptive functioning. It also provides accurate, rather than proxy, information. This is particularly important given the necessity in some cases of different carers providing information at baseline and follow-up (due to staff-turnover, which is unavoidable in research studies with this population), who may have different perceptions/depth of information regarding the person they support. A limitation in interpreting the findings is, however, the small sample size.

The results show that further study of Aβ42/Aβ40 as a potential biomarker is indicated in a larger sample.

Recruiting 60 people to the study was not feasible. Due to the Down syndrome population age distribution, it would have been considerably easier to recruit a larger number had we lowered the entry age to, e.g. 40 or 45, but given the much lower dementia incidence rate at these different ages, it would have required a much longer follow-up time to demonstrate cognitive decline, and hence would not have been affordable as a pilot study. We excluded people who had already acquired dementia, as whilst simvastatin might be beneficial for them, we hypothesised it more likely to be effective in the earlier stages of amyloid deposition.

The prevalence of Down syndrome has varied over time due to increasing life expectancy [61], access to treatments for congenital heart defects with improved surgical techniques and post-operative care [62], increasing maternal age at birth [61], and rates of termination of pregnancy. There is now widespread availability of, and improved and improving methods of antenatal screening for Down syndrome which initially led to an increase in terminated pregnancies [61, 63]; but there are also changing public attitudes, with growing acceptance and integration of disabled people in society. There is some evidence to suggest that the number of people born with Down syndrome is now increasing [64]. A recent study reported Down syndrome in 1.2 of 1000 pregnancies, of which 78.1 % were live births. Survival rate at 1 year for live-births in 1995–1999 was 91.6 %, and 85 % are estimated to survive to 10 years [65]. The proportion of people with Down syndrome reduces in older cohorts [66]. These factors interplay to affect prevalence: in the UK, adult prevalence is estimated at 6 per 10,000 general population [53, 67], but will rise with increasing longevity. At present, there are approximately 30,000 adults with Down syndrome in the UK, of whom about 7500 are aged over 50. Hence, dementia is currently an important healthcare issue, and this will increase to be more important in the future in this growing population. Strategies for prevention and treatment are, therefore, urgently needed: this feasibility study is a very important first step towards this.

## Conclusions

Dementia is a highly disabling, progressive disorder culminating in premature death. It has a negative impact on the adult with dementia, their family and friends, leads to increasing health and social care resources being required, and has a major societal and economic cost. Hence, preventative measures are urgently needed for adults with Down syndrome, and it is crucially important that trials with, and for, them are undertaken. If statins are effective, there is a case for their routine prescription for all adults with Down syndrome. Simvastatin is a very cheap intervention, at £1.23 per 28 tablets (US$1.86; 1.67 €) according to the *British National Formulary*.

A full-scale RCT of simvastatin for the primary prevention of dementia in older adults with Down syndrome is feasible, despite the numerous challenges it poses. This pilot study has provided essential information to enable the design and implementation of a full-scale RCT. It has also highlighted considerable need for publicity and education on the reasons for, and benefits of, conducting RCTs with this population.

This study provides information that is also likely to benefit other future clinical studies with this population. It provides an excellent examples of Cooksey type II translational research (a recognised research gap), inter-sectoral collaboration, novel case identification, and instrument technology development.

## Additional files

Additional file 1: CONSORT 2010 checklist. (DOC 218 kb) Additional file 2: TOP-COG cognitive assessment tasks. (DOCX 15 kb) Additional file 3: CONSORT 2010 flow diagram. (DOC 48 kb) Additional file 4: Illustrative quotes from the nested qualitative study. (DOCX 16 kb)

## Abbreviations

Aβ: amyloid β
ABS: Adaptive Behaviour Scale – Residential and Community score
*APOE*: apolipoprotein E
APP: amyloid precursor protein
CANTAB: Cambridge Neuropsychological Test Automated Battery
CI: confidence interval
EQ-5D: EuroQol 5-Dimension Questionnaire
HPS: Heart Protection Study
ISRCTN: International Standard Randomised Controlled Trial Number
LDLR: low-density lipoprotein receptor
NADIID: Neuropsychological Assessment of Dementia in Intellectual Disabilities
PROSPER: Prospective Study of Pravastatin in the Elderly at Risk
RCB: Robertson Centre for Biostatistics
RCT: randomised controlled trial
