# Supplementary material for: Towards onset prevention of cognition decline in adults with Down syndrome (The TOP-COG study): A pilot randomised controlled trial
Source: Trials. 2016 Jul 29;17:370. doi: 10.1186/s13063-016-1370-9 (PMC4966871; doi:10.1186/s13063-016-1370-9)
Supplement: Additional file 2: — TOP-COG cognitive assessment tasks. (DOCX 15 kb) [file 13063_2016_1370_MOESM2_ESM.docx]

**Additional file 2. TOP-Cog cognitive assessment tasks**

We investigated eight tests in memory, attention, and executive functioning domains:

- Memory for Objects from the NADIID battery,
- Selective Attention Cancellation Task,
- Pattern Recognition Memory from the Cambridge Neuropsychological Test Automated Battery (CANTAB),
- Cats and Dogs test,
- Tower of London Test (adapted for intellectual disabled adults),
- Cued Recall Test,
- Category fluency,
- Story Recall (adapted from the Rivermead Behavioural Memory Test for Children).

| **Task** | **Task Description** |
| --- | --- |
| NADIID Memory for Objects | The participant is asked to name objects which they are shown and then hidden from view. She/he is first shown two everyday objects (e.g. key, pencil) and asked to name them. One object is then covered with a box and the participant is asked to recall what object has been covered. This procedure is then repeated a second time. There are subsequently two trials following the same procedure with 3, 4, 5 and 6 objects. |
| Selective Attention Test | Participants are presented with a 21.6 cm x 27.9 sheet of paper composed of an array of black and white line drawings and are required to detect and cross-out each occurrence of a target item. Each page contained 16 copies of 4 different images arranged randomly in 8 rows with 8 items per row. Each row of drawings contains two copies of each image. Measures are: (a) the time to completion measured in seconds, (b) the number of targets crossed-out (*hits*), and (c) the number of errors made. A composite score based on these three measures is also completed. |
| Pattern Recognition Memory | The subject is presented with a series of 12 visual patterns, one at a time, in the centre of a computer screen. These patterns are designed so that they cannot easily be given verbal labels. In the recognition phase, the subject is required to choose between a pattern they have already seen and a novel pattern. In this phase, the test patterns are presented in the reverse order to the original order of presentation. This is then repeated, with 12 new patterns. |
| Cats and Dogs test | This task is a Stroop-type task which does not require reading, but aims to test the ability to inhibit an established prepotent response. It involves the participant looking at a sequence of images of either a cat or a dog. In the first trial they must say aloud cat or dog according to the picture. In the second trial the participant must say the opposite (i.e. say cat when they see a dog and dog when they see a cat). Scoring is in terms of errors and time to completion for each trial. |
| Tower of London | The Tower of London test consists of a set of three pegs on a wooden base and three different coloured balls which can be moved on the pegs to produce different end-goal states that require different numbers of minimum moves to be made from a start-state. The test starts with a minimum one-move problem and ends with a six-move problem. Each problem level has one problem i.e. one two-move problem, one three-move problem, etc. There are three trials per problem, scored as three points if correct solution in minimum moves on ﬁrst trial, two points if correct solution in minimum moves on second trial and one point if correct solution in minimum moves on third trial. |
| Cued Recall Test | 12 line drawings shown in groups of four. Participant asked to say which is the animal, which is the fruit etc and name object. Pictures are removed and participant asked to recall the four items. Up to three learning trials are used and then after learning trials for each of the three sets of four objects are complete, there is an immediate free recall trial (try to remember all of the objects you have just seen) followed by a cued recall condition (what was the animal, what was the fruit). Three test trials are completed. |
| Category Fluency | This category Fluency task will involve asking participants to say aloud as many animals they can in one minute. |
| Tell a story | A short story comprising 31 idea units is read aloud. The participant is asked to recall as much of the story as possible. They are then asked a series of 10 questions relating to elements in the story. |
