# Supplementary material for: Towards onset prevention of cognition decline in adults with Down syndrome (The TOP-COG study): A pilot randomised controlled trial
Source: Trials. 2016 Jul 29;17:370. doi: 10.1186/s13063-016-1370-9 (PMC4966871; doi:10.1186/s13063-016-1370-9)
Supplement: Additional file 3: — CONSORT 2010 flow diagram. (DOC 48 kb) [file 13063_2016_1370_MOESM3_ESM.doc]

**
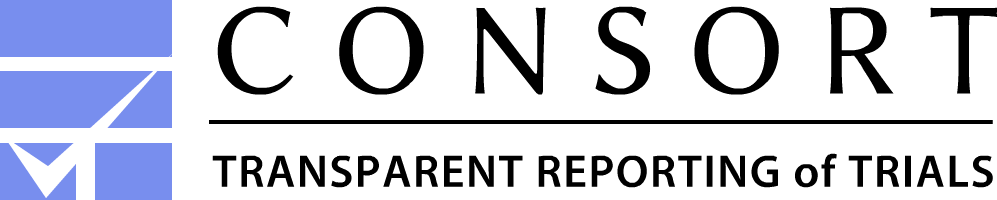
**

**CONSORT 2010 Flow Diagram**

**Allocation**

**Analysis**

**Follow-Up**

**Enrollment**

Assessed for eligibility (n=41)

Excluded (n=20)

♦  Not meeting inclusion criteria (n=16)

♦  Participation declined (n=3)

♦  Died (n=1)

Analysed (n=7)
♦ Excluded from analysis (n=0)

Lost to follow-up (unwilling to continue=3) (n=3)

Discontinued intervention (carer concerned about drug=3; changed mind=1) (n=4)

Allocated to simvastatin 40mg OD (n=10)

♦ Received allocated intervention (n=10)

♦ Did not receive allocated intervention (n=0)

Lost to follow-up (unwilling to continue=2) (n=2)

Discontinued intervention (ill-health=2; GP prescribed a statin=1; changed mind=1) (n=4)

Allocated to placebo (n=11)

♦ Received allocated intervention (n=11)

♦ Did not receive allocated intervention (n=0)

Analysed (n=9)
♦ Excluded from analysis (n=0)

Randomized (n=21)
