# Supplementary material for: Towards onset prevention of cognition decline in adults with Down syndrome (The TOP-COG study): A pilot randomised controlled trial
Source: Trials. 2016 Jul 29;17:370. doi: 10.1186/s13063-016-1370-9 (PMC4966871; doi:10.1186/s13063-016-1370-9)
Supplement: Additional file 4: — Illustrative quotes from the nested qualitative study. (DOCX 16 kb) [file 13063_2016_1370_MOESM4_ESM.docx]

# **Additional file 4. Illustrative quotes from the nested qualitative study**

**The Value of Research**

Quote 1: *Well it’s the only way we’re going to find out what’s going on. It’s important…I think it’s a great idea so yeah research is important.*

Quote 2: *I don’t think I can express how important I think research is. I used to work in trials many years ago before I got sick so I understand why we need them. How can you find out new cures or treatments?*

Quote 3: *It’s the only way we’re going to learn anything isn’t it?*

Quote 4: *I think they’re [research studies] worthwhile and if you don’t do them then how do we progress? How do we find out what works and what doesn’t work?*

Quote 5: *I think they’re good most of the time. If they’re safe then a good idea [sic].*

Quote 6: *D’nae know much about them to be honest.*

Quote 7: *I don’t really know enough about them. I don’t hear very much in the news or whatever so I’m not really sure.*

**Information sheets are useful, and an additional resource**

Quote 8: *[Information sheets] were easy to understand…yeah you can get too much information but that was just enough.*

Quote 9: *No there’s nothing in the information sheets that put me off. There was just enough information.*

Quote 10: *Fine. Enough information…not easy-read though.*

Quote 11: *No there was enough [information]…we saw them as an added thing to guide us.*

Quote 12: *…We thought well actually we would be interested if it was explained to us about the chromosome extra [sic] that he’s got and that causes more of the stuff…*

Quote 13: *I think I would have just participated anyway.*

Quote 14: *No no I was looking to participate anyways. So I would have done it no matter how we had got the information.*

Quote 15: *No it wouldn’t have changed anything.*

Quote 16: *Nah that doesn’t really matter to me. If I don’t like something I don’t like it.*

**Preventing dementia is the primary motivation for deciding to take part**

Quote 17: *Any carer of a person with Down’s Syndrome in their fifties it’s [dementia] probably their biggest fear.*

Quote 18: *I think for us it’s always been a fear for us. Eh the knowledge-the knowledge that we got was normally beyond fifty it’s almost all people with Downs have this and we kinda knew where we were and we’ve been taking care of him since he was forty we had to be prepared as a family…*

Quote 19: *I don’t want him getting dementia.*

Quote 20: *So because [dementia] it is our one worry about his future why would we no [take part]? Makes sense you know? It’s the same reason we got him checked every so often it was for his sake we were doing the right thing at the right time because you just never know…so we want to get it right for him you know? We’ve been left a responsibility so why would we no?*

Quote 21: *Because I don’t want dementia I don’t want Alzheimer’s. So the only way I can keep [indistinct] is by doing this.*

Quote 22: *You know if there’s anything I can do to prevent it [dementia] then I’m going to do it.*

Quote 23: *I think because it [the RCT] was about dementia at this stage in KS’s life…is something we would grab on to…The fact you know we’ve got a sister with Down’s Syndrome…and we were worried about dementia…[taking part] is a bit of a no-brainer.*

Quote 24: *Well I asked him if he wanted to do it [take part] and this test would help some of his friends maybe that would (sic) have dementia and see I maybe felt it help someone else so he was quite happy to do it after that.*

Quote 25: *Well if it’s going to help or help someone else then whatever helps you know?*

**Taking tablets is the most common reason for declining to participate**

Quote 26: *I mean yeah if a doctor tells me he needs them then fine but d’nae want him taking them for nae reason pal… Taking a drug, that’s a big problem for me. I feel like you’re testing something out on my brother you know? How do you know what you’re giving him?*

Quote 27: *I am on some tablets but I don’t really like them. I’d rather not be on tablets...I don’t like drugs.*

Quote 28: *D’nae like giving her a tablet when you don’t know it works…Could be harmful.*

Quote 29: *I would worry about side effects…I used to take a statin and I had bad side effects so that would really put me off having her on the study ‘cause she could have them too and then what’s the point of that you know?*

Quote 30: *…they [statins] could be harmful…and you know, she’s got Down’s so feels like you’re using her a bit…*

Quote 31: *I don’t like that.*

Quote 32: *That seems unfair. You take a pill and then find out there’s nothing in it so you’ve done it for nothing. How is that good?*

**Tablets are okay so long as they are necessary**

Quote 33: *I took that many myself ha ha so I’m diabetic so there’s quite a lot of tablets but I uh personally if that’s what keeps you going then you’ve no much choice have yeh?*

Quote 34: *Well less is best is my policy but I’m not adverse to it if you need it you have it.*

Quote 35: *…because she’s on medication anyway it was just a matter of taking them.*

Quote 36:  *I take-I take a lot of tablets so I don’t have a problem taking tablets. Certainly if it helps then why not? As long as you way up the side effects.*

Quote 37: *Eh uh it was a bit concerning initially [not knowing if on a statin or a placebo] but only because we knew statins had side effects so we were looking for stuff because we knew we had to interpret it for him*

Quote 38: *I mean I think, I think it’s necessary…It’s eh annoying because a part of me thinks well if she’s on the statin and it’s going to help it’s not helping ha ha or is it?...But I mean I think you’ve just got to decide that you’re a part of the and you’ll get the results…the doctor gives you tablets…you get an antibiotic I mean you don’t even know if that antibiotic is going to work on you you just don’t know because everyone reacts differently so you’ve just got to hope you uh trust the doctor and we’ve trusted you and we just decided that we’ll see what it’s like at the end of the study…And then if she’s not [on the statin] and it’s worked well then she can go on them.*

Quote 39: *I don’t know but I’d rather you told me…I want to know now. The only thing I want to know as well is who got the placebo and whose got the real thing? I don’t want to go on the kid on one I want to go on the real one.*

Quote 40: *Well it’s what we signed up for so…it would be nice to know but that’s part of the terms and conditions and I do understand about blind testing. I’ve done quite a lot of trials over the years…but no don’t have a problem with that. It would have been nice to know and it will be nice to know.*

**Taking part in the RCT is a positive experience**

Quote 41: *It was nice for you to come and visit us. You’ve always gotten on with KS well even when she’s been a bit funny sometimes…I’ve been able to ask you other questions I’ve unloaded on you ha ha you’ve been a good sounding board about her behaviour changes and I found the interviews and tests for KS quite fascinating.*

Quote 42: *…wasn’t a hard study to take part in and looking forward to the results.*

Quote 43: *I liked the fact that there was the personal stuff you know and then there was you in particular…it was all very professional it was all very sensitive. No there’s nothing not to like about it to be honest.*

Quote 44: *I found them amazing…it was just interesting with AC because I think he never-every day is a school day and I learned things about AC doing them. Thank you for letting him be on the trial because it means such a lot to us to be able to know how AC ticks you know?*

Quote 45: *…I’m glad we did it and I’ve learnt a lot about HM…*

Quote 46: *I thought it was quite unobtrusive and I think you were very thorough when doing the blood tests for various things you know?*

Quote 47: *I think what I’ve liked is that its been simple and straightforward but not too intrusive. It hasn’t been you know if you’d been doing more more (sic) it would’a been ‘oh no here’s more of this top cog stuff I’ve got to do here’ whereas it- you know it wasn’t – I knew what was happening that it didn’t impact my everyday life.*

Quote 48: *Great. Very interesting…yeah I found them quite interesting because I like to know how she’s getting on…as well as it’s interesting to see ‘em. You know quite reassuring when you’re sitting here trying to do them yourself. I was gonna try start to find out about it. I didn’t know where to start.*

**Lack of accessible information on Down syndrome and dementia**

Quote 49: *Through the study we’ve found out more information about dementia and Down syndrome that we didn’t have before you know? I don’t know how else we would find out about it you know? Probably would have started with the GP.*

Quote 50:  *…but I think the fact that it was about dementia at this stage in KS’s life is something we would grab on to…you know we’re looking for information on that. It’s obviously something we’re very worried about.*

Quote 51: *This will be a bit of a start on dealing with dementia because they’re early onset really compared to everyone else…and I never really knew about that until 3 or 4 years ago when he was already 53.*

Quote 52: *No I’d never heard of it [research]. You very rarely ever hear about research they’re doing in Down syndrome. I like getting-I like information I want to know as much as possible about something you know? I mean I remember hearing years ago there was something they were going to do to help people with Down’s syndrome and then you never hear about it again.*

Quote 53: *Well you don’t get told…oh no no one ever discussed this with me and my mother certainly didn’t.*

Quote 54: *In fact a couple of the carers at his centre I spoke to, they didn’t know it [about risk of dementia].*
